# Effects of dietary rumen-protected choline supplementation on colostrum yields, quality, and choline metabolites from dairy cattle

**DOI:** 10.3168/jdsc.2021-0192

**Published:** 2022-04-26

**Authors:** T.H. Swartz, B.J. Bradford, O. Malysheva, M.A. Caudill, L.K. Mamedova, K.A. Estes

**Affiliations:** 1 Department of Animal Science, Michigan State University, East Lansing 48824; 2 Division of Nutritional Sciences, Cornell University, Ithaca, NY 14853; 3 Balchem Corporation, New Hampton, NY 10958

## Abstract

Colostrum is a critical nutrient source that provides passive immunity to dairy calves. Choline is a trimethylated molecule that is frequently supplemented in the diet to periparturient dairy cows to support postpartum health and performance. Whereas choline and its metabolites have been characterized in milk, the effects of dietary rumen-protected choline (RPC) supplementation on choline metabolites in colostrum from dairy cattle have yet to be explored. Therefore, the objective of the present study was to assess the effects of dietary supplementation and dose of RPC on colostrum yields, quality, and choline metabolites. Parous Holstein cows were blocked by calving month and randomly assigned within block to receive 45 g/d (20.4 g/d of choline ions) of RPC (CHOL45, n = 22), 30 g/d (13.6 g/d of choline ions) of RPC (CHOL30, n = 20), or no RPC (control, n = 19) starting 24 d before expected calving. The effects of dietary supplementation and dose of RPC were assessed on colostrum yields, component yields, somatic cell score (SCS), quality (as assessed by Brix), and choline metabolites. Data were analyzed using a linear mixed model with the fixed effects of treatment, parity, and the 2-way interaction and the random effect of block. Regardless of dose, dietary RPC supplementation increased colostrum yields and protein yields. No effects of dietary RPC supplementation were found on colostrum component percentages, SCS, or colostrum quality. For choline metabolites, treatment interacted with parity for phosphocholine where colostrum from second-parity CHOL45 and CHOL30 cows had greater concentrations of phosphocholine than colostrum from second-parity control cows, but no treatment effect was seen in the colostrum from 3+ parity cows. Dietary choline supplementation, regardless of dose, increased trimethylamine *N*-oxide concentrations. Dietary choline supplementation did not affect the concentrations of choline, betaine, glycerophosphocholine, sphingomyelin, phosphatidylcholine, or total choline in colostrum. In conclusion, dietary choline supplementation increased phosphocholine concentrations in colostrum from second-parity cows, enhanced trimethylamine *N*-oxide concentrations, and increased colostrum yields without affecting colostrum quality.

## Highlights

• Dietary choline supplementation increased colostrum yields from Holstein cows.
• Choline supplementation increased phosphocholine concentrations from second-parity cows.
• Choline requirements might be greater in 3+ parity cows as compared with second parity.
• Choline supplementation increased trimethylamine N-oxide concentrations in colostrum.

## Graphical Abstract

**Figure:**
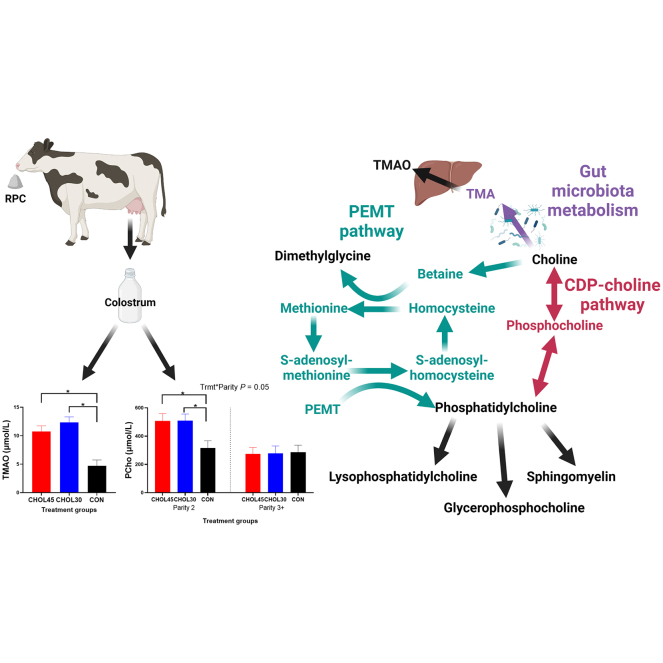
**Summary:** We assessed the effects of dietary choline supplementation on colostrum yields, quality, and choline metabolites from dairy cattle. Dietary choline supplementation increased the amount of colostrum produced without affecting the concentration of antibodies. Furthermore, dietary choline supplementation enhanced phosphocholine concentrations in colostrum from cows calving for the second time but had no effect on phosphocholine concentrations in colostrum from older cows. Finally, dietary choline supplementation increased concentrations of trimethylamine N-oxide, which is a choline metabolite derived from gut microbial metabolism.

Choline is a bioactive micronutrient that serves as a precursor to numerous molecules. Water-soluble choline metabolites include betaine (methyl donor), acetylcholine (neurotransmitter), glycerophosphocholine (**GPC**; osmolyte), and phosphocholine (**PCho**; intermediate pool of intracellular choline, Zeisel and Blusztajn, 1994). Lipid-soluble choline metabolites include phosphatidylcholine (**PC**) and sphingomyelin (**SM**), which are structural components of cell membranes and have cell signaling properties, and partial hydrolysis of PC will yield the metabolite lysophosphatidylcholine (Zeisel and Blusztajn, 1994). The fate of choline depends mainly on 2 pathways, (1) the cytidine diphosphate (**CDP**) choline pathway and (2) the phosphatidylethanolamine *N*-methyltransferase (**PEMT**) pathway. For the CDP-choline pathway, PCho is synthesized from choline and PC is subsequently produced from PCho. However, PC can also be derived by methylating phosphatidylethanolamine via the PEMT pathway. Choline can be converted irreversibly to betaine via the enzyme choline dehydrogenase. Using betaine as the methyl donor, the PEMT pathway methylates homocysteine to form methionine, which is then converted to S-adenosylmethionine via methionine adenosyl transferase. S-Adenosylmethionine donates methyl groups to phosphatidylethanolamine to synthesize PC (Zeisel and Blusztajn, 1994). Either GPC or SM can be synthesized from PC. Aside from those metabolites, acetylcholine is synthesized from choline via choline acetyltransferase. Finally, another metabolite of recent interest is trimethylamine *N*-oxide (**TMAO**), which is derived from microbial metabolism of choline to trimethylamine in the gut, which is then oxidized by flavin-containing monooxygenases in the liver (Wang et al., 2011).

Choline may be supplemented in the rumen-protected (**RPC**) form to support performance in postpartum dairy cows. Indeed, it has long been speculated that choline is a limiting nutrient in high-yielding dairy cows due to the immense output of methylated compounds in milk (Pinotti et al., 2002), and in a recent meta-analysis, dietary RPC supplementation during the peripartum period increased milk yield by almost 2 kg/d (Arshad et al., 2020). Although it is well established that dietary choline supplementation enhances milk yield of postpartum dairy cows, little is known about its effect on colostrum, a critical nutrient source for newborn calves to provide passive immunity through the absorption of immunoglobins across the gut. Moreover, even less is known about dose responses of RPC. As such, our objective was to assess the effects of dietary supplementation and dose of RPC to periparturient dairy cows on colostrum yields, quality, and choline metabolites. We hypothesized that dietary RPC supplementation would enhance colostrum yields and quality and increase the concentrations of choline metabolites in a dose-dependent manner.

All experimental procedures were conducted at the Michigan State University Dairy Cattle Teaching and Research Center in East Lansing, Michigan, from January through August 2021 in accordance with a protocol approved by the Michigan State University Institutional Animal Care and Use Committee (PROTO202000184).

Close-up dry Holstein parous cows (n = 67) were blocked by expected calving month and randomly assigned within block to receive 1 of 3 treatments. Cows with nonfunctional mammary quarters were not enrolled. Dietary treatments were topdressing of 45 g/d of RPC (**CHOL45**, n = 23), 30 g/d of RPC (**CHOL30**, n = 22), or no RPC (**CON**, n = 22) starting approximately 24 ± 3 d before expected calving until 21 d postpartum. The 30 and 45 g/d doses provided 13.6 and 20.4 g/d of choline ions, respectively. The RPC, which was provided in kind (Balchem Corporation, New Hampton, NY), was mixed with ground corn by the research staff and topdressed for a total weight of 150 g/d, and CON cows received 150 g/d of ground corn. The RPC product used in this study is not currently commercially available. The supplement consisted of a choline chloride core and a lipid coating, and the encapsulation technology used was similar to that of all commercially available Balchem Corporation products. Rumen disappearance was determined using an in situ procedure by a third-party certified commercial laboratory. Over a 12-h time frame, this dietary RPC supplement had 74.9% choline chloride remaining.

Close-up dry cows were housed in freestalls with rubber mattresses that were bedded with sawdust. Cows were fed once daily in the morning, which was when dietary treatments were applied. Just before the delivery of the TMR, headlocks were set to lock up cows such that as the feed was being dispensed from the mixer, cows would be caught in the headlocks. Once all cows were restrained, individual treatments were provided. Approximately 45 min later, the TMR was inspected to ensure that the supplement had been consumed by each cow. All cows consumed at least some of their daily supplement; however, there were a few rare instances where a cow did not consume the entire supplement. If this happened, the remaining supplement was removed from the feed bunk to ensure that other cows did not consume it. Cows were then released from the headlocks. The close-up diet (Table 1) was formulated using AMTS software (Groton, NY) to meet nutritional requirements of a 620-kg cow in late gestation consuming 12.7 kg of DM/d; supplemental rumen-protected Met was included to evaluate choline effects in a Met-sufficient context. Feed samples of the close-up TMR were taken weekly, composited by month, and were evaluated using near-infrared spectroscopy (Cumberland Valley Analytical Services). The ingredients and chemical composition of the diet are provided in Table 1.

**Table 1:** Ingredients and chemical composition of the close-up diet

Item, DM basis (except where noted)	Close-up diet
Ingredient (%)	
Corn silage	39.7
Grass hay	27.9
Canola meal	10.0
Soybean meal	8.2
Corn grain, finely ground	4.1
Acidified soybean meal^1^	2.9
Soybean hulls, pelleted	1.8
Calcium carbonate	1.5
Calcium sulfate	1.0
Magnesium sulfate	0.82
Mineral product^2^	0.72
Protein blend^3^	0.54
Vitamin and trace mineral supplement^4^	0.32
Dicalcium phosphate	0.29
Tallow	0.21
Magnesium oxide	0.071
Rumen-protected methionine^5^	0.034
Nutrient (mean ± SD)	
NE_L_ (Mcal/kg)	1.44 ± 0.033
CP (%)	13.2 ± 1.4
MP (g/d; model estimate)	1,218
Metabolizable Met^6^ (g/d; model estimate)	29
NDF (%)	43.5 ± 3.4
ADF (%)	29.2 ± 2.02
Lignin (%)	5.0 ± 0.32
Starch (%)	16.9 ± 1.03
Ether extract (%)	3.1 ± 0.12
Ash (%)	8.2 ± 0.18
Ca (%)	1.1 ± 0.11
P (%)	0.36 ± 0.033
Mg (%)	0.46 ± 0.038
K (%)	1.05 ± 0.019
Na (%)	0.20 ± 0.032
Mn (mg/kg)	80.2 ± 13.0
Zn (mg/kg)	62 ± 7.0
Cu (mg/kg)	13 ± 3.0
Fe (mg/kg)	305 ± 31
DCAD (mEq/kg)	−123

1 SoyChlor, Landus Cooperative.

2 Min-Ad Inc.

3 Caledonia Pass, Caledonia Farmers Elevator Co.

4 The premix contained 30.5% Na, 7,567 mg/kg Zn, 3,888 mg/kg Mn, 961 mg/kg Cu, 105 mg/kg Co, 125 mg/kg I, 67 mg/kg Cr, 74 mg/kg Se, 1.93 kIU/g vitamin A, 1.11 kIU/g vitamin D_3_, and 29.66 IU/g vitamin E.

5 Smartamine M, Adisseo North America.

6 CPCNS model predictions included a Lys:Met ratio of 2.86 and a metabolizable Met:metabolizable energy ratio of 1.06 g/Mcal.

Colostrum was harvested within 6 h of calving and weighed by the farm staff, who were blinded to treatment allocation. Six colostrum samples were not collected resulting in 61 total colostrum samples (CHOL45, n = 22; CHOL30, n = 20; CON, n = 19). Two aliquots of colostrum were collected per cow. One 50-mL aliquot was collected in a sealed tube with a preservative (bronopol) and stored at 4°C for colostrum component and SCC analyses (Bentley FTM/FCS, Bentley Instruments Inc.) by the Michigan Dairy Herd Improvement Association (Central Star DHI). Another aliquot was collected in a 50-mL conical tube and stored at −80°C for the purpose of quantifying choline metabolites. Before statistical analysis, SCC was transformed to SCS [SCS = log_2_(SCC/100,000) + 3]. Colostrum quality (Brix units, %) was assessed using a digital refractometer (Digital Dairy Refractometer, MISCO Refractometer). Choline, betaine, methionine, and TMAO were extracted from 50 µL of colostrum and quantified in duplicate by liquid chromatography–tandem mass spectrometry (**LC-MS/MS**) with some minor modifications to the methods outlined in Holm et al. (2003) and Yan et al. (2012). Similarly, PC, SM, GPC, and PCho were extracted from 100 µL of colostrum and quantified in duplicate by LC-MS/MS according to the method of Koc et al. (2002) with some modifications (Yan et al., 2013).

Linear mixed models were conducted using PROC GLIMMIX (SAS 9.4, SAS Institute Inc.). The model included the fixed effects of treatment, parity (2 and 3+), and the 2-way interaction, with the random effect of block. Covariates included genetic traits (PTA for milk, fat, protein, and SCS in their respective models) and BCS recorded just before applying treatment (−24 d) as well as the interaction of the covariates with treatment; however, none of these were significant. Backward elimination was used to remove nonsignificant terms until all variables in the model had a *P* ≤ 0.05 except for treatment, which was forced into the model. Treatment least squares means were separated using the PDIFF statement with a Tukey adjustment. In all models, residuals were evaluated for normality and outliers (PROC UNIVARIATE). If an outcome variable was nonnormally distributed, the natural logarithmic transformation was used. Significance was declared at *P* ≤ 0.05.

Colostrum yields and component yields were affected by treatment (all *P* ≤ 0.01; Table 2). Specifically, CHOL45 and CHOL30 cows produced 2.5 and 2.9 kg more colostrum than CON, respectively (CHOL45 vs. CON, *P* = 0.05; CHOL30 vs. CON, *P* = 0.02). No effect of treatment was found on colostrum fat, protein, or lactose percentages (all *P* ≥ 0.21). Nevertheless, CHOL30 cows produced more colostrum fat, protein, and lactose yields (all *P* < 0.01) relative to the CON. Cows in the CHOL45 group produced more colostrum protein (*P* = 0.05), but no difference was found in lactose (*P* = 0.06) or fat yields (*P* = 0.22) when compared with the CON. Although treatment influenced colostrum yields, it did not affect colostrum quality as assessed by Brix refractometry (*P* = 0.29) or SCS (*P* = 0.09).

**Table 2:** Effects of prepartum dietary supplementation of rumen-protected choline at 45 g/d (CHOL45, n = 22), 30 g/d (CHOL30, n = 20), or no supplementation (CON, n = 19) on colostrum yield and quality

Item	LSM			SE	*P*-value	
	CHOL45	CHOL30	CON		Treatment	Parity
Colostrum yield (kg)	5.9^a^	6.3^a^	3.4^b^	0.83	0.01	NS
Fat (%)	4.5	4.6	3.8	0.66	0.29	NS
Protein (%)	11.5	12.0	11.4	0.61	0.76	0.04
Lactose (%)	3.1	3.2	2.9	0.12	0.21	NS
Fat yield (kg)	0.24^ab^	0.32^a^	0.15^b^	0.051	0.01	NS
Protein yield (kg)	0.68^a^	0.78^a^	0.38^b^	0.10	<0.01	NS
Lactose yield (kg)	0.18^ab^	0.21^a^	0.10^b^	0.027	<0.01	NS
SCS^1^	6.0	5.9	6.9	0.40	0.09	<0.01
Brix (%)	22.3	22.3	23.8	0.83	0.29	<0.01

a,b Within a row, means with different superscripts differ (*P* ≤ 0.05).

1 SCS: log_2_(SCC/100,000) + 3.

Of all choline metabolites detected, GPC was found in the greatest quantity in colostrum followed by SM, PCho, PC, betaine, and choline (Table 3). Relatively small quantities of TMAO were found, whereas acetylcholine and lysophosphatidylcholine were not detected. Treatment did not influence the concentrations of choline, betaine, GPC, SM, PC, or total choline in colostrum (all *P* ≥ 0.13); however, treatment did affect the yields of all aforementioned metabolites (all *P* ≤ 0.03). Colostrum from CHOL30 cows had greater choline, betaine, GPC, SM, PC, and total choline yields than colostrum from CON cows (all *P* ≤ 0.03). Relative to the CON, colostrum from CHOL45 cows had greater quantities of choline, betaine, GPC, and total choline (all *P* ≤ 0.05), but no difference was found for PC (*P* = 0.28) and SM (*P* = 0.06). Methionine was found in relatively small quantities in colostrum and dietary RPC supplementation did not influence its concentrations (*P* = 0.06) or yields (*P* = 0.55).

**Table 3:** Effects of prepartum dietary supplementation of rumen-protected choline at 45 g/d (CHOL45, n = 22), 30 g/d (CHOL30, n = 20), or no supplementation (CON, n = 19) on the concentrations and yields of choline metabolites in colostrum

Item	LSM			SE	*P*-value		
	CHOL45	CHOL30	CON		Treatment (T)	Parity (P)	T × P
Metabolite concentration (μmol/L)							
Choline^1^	5.1	5.2	5.1	0.13	0.84	0.05	NS
Betaine	246	250	214	21	0.13	0.04	NS
Trimethylamine *N*-oxide	10.7^a^	12.3^a^	4.7^b^	0.99	<0.0001	NS	NS
Glycerophosphocholine	4,013	3,958	3,440	219	0.14	NS	NS
Sphingomyelin	523	536	507	48	0.82	NS	NS
Phosphatidylcholine^1^	5.7	5.8	5.8	0.13	0.79	NS	NS
Phosphocholine	391	393	301	38	0.09	<0.0001	0.05
Total choline^2^	5,472	5,417	4,815	285	0.15	NS	NS
Methionine	5.2	6.1	9.2	1.2	0.06	0.01	NS
Metabolite yield (μmol/cow)							
Choline	963^a^	1,000^a^	483^b^	169	0.02	NS	NS
Betaine	1,401^a^	1,492^a^	755^b^	211	<0.01	0.05	NS
Trimethylamine *N*-oxide	57.4^a^	75.8^a^	16.1^b^	9.7	<0.0001	NS	NS
Glycerophosphocholine	23,869^a^	24,262^a^	11,734^b^	3,715	<0.01	NS	NS
Sphingomyelin	3,123^ab^	3,274^a^	1,694^b^	509	0.02	NS	NS
Phosphatidylcholine	1,729^ab^	2,140^a^	1,181^b^	297	0.03	NS	NS
Phosphocholine	2,579^a^	2,958^a^	1,106^b^	527	0.01	0.01	NS
Total choline^2^	32,645^a^	33,824^a^	16,116^b^	5,118	<0.01	NS	NS
Methionine	20.5	25.3	22.2	3.8	0.55	<0.01	NS

a,b Within a row, means with different superscripts differ (*P* ≤ 0.05).

1 Data are presented using a natural logarithmic transformation.

2 Sum of all choline-containing biomolecules (choline, glycerophosphocholine, sphingomyelin, phosphatidylcholine, and phosphocholine).

For PCho concentrations, the effect of treatment was dependent on parity (interaction, *P* = 0.05), where colostrum from second-parity cows receiving either 45 or 30 g of RPC had greater concentrations of PCho than colostrum from CON cows (CHOL45 vs. CON, 508 ± 52 vs. 317 ± 51 µmol/L, *P* = 0.02; CHOL30 vs. CON, 508 ± 48 vs. 317 ± 51 µmol/L, *P* = 0.01), but no treatment effect was seen in 3+ parity cows (273 ± 46, 278 ± 52, and 286 ± 50 µmol/L for CHOL45, CHOL30, and CON, respectively; all comparisons, *P* ≥ 0.98). Treatment influenced the yields of PCho (*P* = 0.01), where colostrum from both CHOL45 (*P* = 0.05) and CHOL30 (*P* = 0.01) had greater quantities than colostrum from CON. Treatment was highly significant for TMAO concentrations (*P* < 0.0001) and yields (*P* < 0.0001), where colostrum from both CHOL45 and CHOL30 cows had greater concentrations (both comparisons, *P* < 0.0001) and yields (both comparisons, *P* < 0.01) than CON.

The primary objectives of our study were to assess the effects of dietary supplementation and dose of RPC on colostrum yields, quality, and choline metabolites. Our study demonstrated that dietary RPC supplementation can enhance colostrum yields, as well as increase the concentrations and yields of certain choline metabolites but had no effect on colostrum quality (as assessed by Brix). Moreover, no differences in any outcome were found in colostrum from close-up cows supplemented with 13.6 g/d (CHOL30) as compared with 20.4 g/d of choline ions (CHOL45). As such, our data show a clear effect of dietary RPC supplementation on colostrum yields and choline metabolites with minimal effects due to dose.

Dietary choline supplementation, regardless of the dose, increased yield of colostrum and protein. Whereas past studies have not found an effect of dietary RPC supplementation on colostrum yields (Zenobi et al., 2018; Bollatti et al., 2020), numerous studies have found that dietary RPC supplementation enhanced milk yield by almost 2 kg/d (meta-analysis, Arshad et al., 2020). The effects of dietary choline supplementation on milk yield are dependent on postpartum supply of metabolizable methionine (Arshad et al., 2020); however, the importance of this on colostrum yield is still unknown. Furthermore, a limitation to our study is that prepartum DMI were not measured, which may have explained treatment effects, although this seems unlikely as a meta-analysis found only a small dietary RPC effect on prepartum intakes (0.2 kg/d; Arshad et al., 2020).

One of the concerns about increasing colostrum yield may be the dilution of IgG (Maunsell et al., 1999; Morin et al., 2010; Silva-del-Río et al., 2017). Nevertheless, our study did not find any difference in colostrum quality between treatment groups, similar to the results of Bollatti et al. (2020), although Zenobi et al. (2018) found greater IgG concentrations and total IgG yields. As such, it remains uncertain if dietary RPC supplementation enhances IgG concentrations in colostrum. Our study is the first to characterize choline metabolites in bovine colostrum. The metabolite found in the greatest quantity was GPC followed by SM, PCho, PC, betaine, and choline. In general, the distribution of choline metabolites in bovine colostrum was similar to that found in porcine colostrum, where GPC was also the most abundant (Mudd et al., 2016). Concentrations of choline metabolites change quickly in bovine milk throughout the course of lactation (Artegoitia et al., 2014). Indeed, the choline metabolite profile from colostrum in the present study is substantially different from that of bovine milk (Artegoitia et al., 2014). The biggest differences between our study and Artegoitia et al. (2014) would be that GPC represented almost 75% of total choline moiety in colostrum, whereas in bovine milk, GPC represented less than 10% of choline moiety. In fact, most choline metabolites were PC and PCho in milk, which together represented 60 to 80% of choline moiety depending on the stage of lactation (Artegoitia et al., 2014). As such, it appears that stage of lactation is a critical factor when assessing the distribution of choline metabolites.

Aside from SM and PC, dietary RPC supplementation increased the yields of all other choline metabolites, which was largely a function of increased colostrum yield. More noteworthy, dietary RPC supplementation increased the concentrations of PCho in colostrum from second-parity cows but had no effect on PCho concentrations in colostrum from 3+ parity cows. Phosphocholine is formed by choline kinase and is used as an intermediate pool to synthesize PC (Zeisel and Blusztajn, 1994). An increase in PCho may suggest an enhancement in phosphorylation of choline. Indeed, past research found that dietary RPC supplementation increased hepatic mRNA abundance of the choline kinase A gene (Zhou et al., 2017). With that said, no effect of dietary RPC supplementation was found on total PC, suggesting that downstream reactions from PCho may not have been similarly upregulated, leading to an accumulation of PCho. Nevertheless, it is unclear why this occurred in second-parity but not in 3+ parity cows. One hypothesis is that older cows may have greater choline requirements to preferentially flux more choline through the PEMT pathway to synthesize methionine and subsequently less choline is used via the CDP-choline pathway. In support of this, betaine concentrations were lesser and methionine concentrations were greater in colostrum from 3+ parity cows relative to second-parity cows likely due to enhanced utilization of betaine as a methyl donor to synthesize methionine. Thus, our data provide some evidence of parity preferences in which pathway is used for choline metabolism and support the notion that methionine supply is inadequate in the diet of older cows (Potts et al., 2020).

Dietary choline supplementation enhanced TMAO concentrations, which is derived from gut microbial metabolism of choline into TMA that is oxidized by flavin-containing monooxygenases (Wang et al., 2011). The origin of the precursor of TMAO (TMA) may have been either the rumen or intestine, which is mostly dependent on the degree of ruminal protection. Regardless, TMAO has been associated with cardiovascular disease, diabetes, and cancer in humans, although the direct effects remain unclear (Ufnal et al., 2015). Nevertheless, recent research has pointed to a proinflammatory effect of TMAO using either cell culture methods or mouse models (Sun et al., 2016; Boini et al., 2017); however, it should be noted that these studies used greater TMAO concentrations (at least 3 times more) than the relatively small concentration we found in colostrum. Therefore, we can only speculate on the effect of enhancing TMAO concentrations in colostrum; however, past studies did not reveal an effect on liver health or milk production in dairy cows (Myers et al., 2021).

In conclusion, dietary RPC supplementation enhanced colostrum yields without affecting colostrum quality. Moreover, dietary RPC supplementation increased PCho concentrations in second-parity cows, but not for 3+ parity cows. As such, our study suggests that there may be parity preferences for choline metabolic pathways. Finally, dietary RPC supplementation enhanced TMAO in colostrum. Future studies should assess how altering the concentrations of choline metabolites in colostrum may influence the health and performance of periparturient dairy cows and newborn calves.
